# Digital health maturity models for Human Resources for Health Information Systems in low- and middle-income countries in Africa: A scoping review

**DOI:** 10.4102/jphia.v17i1.2010

**Published:** 2026-09-08

**Authors:** Agbonkhese I. Oaiya, Christmal D. Christmals, Hillary K. Kipruto

**Affiliations:** 1 Centre for Health Professions Education, Faculty of Health Sciences, North-West University, Potchefstroom, South Africa; 2 World Health Organization Regional Office for Africa, Brazzaville, Republic of Congo

**Keywords:** scoping review, health professions education, human resources for health, human resources for health information system, digital health, maturity model, low- and middle-income countries, Africa

## Abstract

**Background:**

Health workforce challenges in Africa are exacerbated by fragmented and underdeveloped Human Resources for Health Information Systems (HRH-IS). While digital health maturity models exist, their applicability to HRH-IS in low- and middle-income countries (LMICs) remains unclear, with limited evidence of context-specific frameworks.

**Aim:**

This scoping review aimed to (1) map digital health and health information system (HIS) maturity models, frameworks, tools and guidelines relevant to HRH-IS, and (2) synthesise key domains to inform the development of a context-appropriate HRH-IS maturity model for African LMICs.

**Setting:**

The study was conducted in low- and middle-income countries in Africa.

**Methods:**

A scoping review was conducted following the Arksey and O’Malley framework and Preferred Reporting Items for Systematic Reviews and Meta-Analyses extension for Scoping Reviews guidelines. PubMed, EBSCO-hosted databases, Google Scholar and grey literature sources, including institutional repositories and global health agency publications were searched. Data were extracted using a structured form and analysed descriptively, with thematic synthesis used to identify transferable domains.

**Results:**

A total of 3128 records were screened, and 41 were included. Only one tool targeted HRH-IS. Most identified frameworks addressed broader digital health or HIS. Six cross-cutting domains were synthesised: (1) governance and leadership; (2) workforce and organisational capacity; (3) financing and sustainability; (4) data architecture and interoperability; (5) data quality and analytics; and (6) equity, trust and system adoption.

**Conclusion:**

There is limited evidence of dedicated HRH-IS maturity models in African LMICs. However, existing digital health and HIS frameworks provide a strong foundation for adaptation.

**Contribution:**

The six synthesised domains offer a preliminary basis for developing and validating a context-specific HRH-IS maturity model to strengthen workforce data systems and support evidence-informed policy.

## Introduction

Health systems in Africa face persistent structural challenges that constrain service delivery and progress towards Universal Health Coverage (UHC). Among these, health workforce shortages, maldistribution and competency gaps remain critical. Estimates suggest a shortfall of approximately 6.1 million health workers across the region, while training capacity remains insufficient to close this gap.^1^ These challenges are further compounded by weak and fragmented Human Resources for Health Information Systems (HRH-IS), which limit the availability of reliable workforce data for planning, policy formulation and performance monitoring.^1^

To address these challenges, countries have adopted various digital health strategies, frameworks and maturity models to guide the design, implementation and evaluation of these technologies in health.^2,3,4^ Examples include the World Health Organization (WHO) Global Strategy on Digital Health, the Digital Health Profile and Maturity Assessment Toolkit (DHPMAT-MM), and the Informatics-Savvy Health Organization (ISHO) framework.^2,3,4^ These tools aim to support structured digital transformation; however, their applicability to specific domains such as human resources for health (HRH) in the low- and middle-income countries (LMIC) context, remains limited.^5^

Among these limitations is the misuse of key concepts and definitions. Technology in health is an emerging field, and there are several overlapping definitions that are used interchangeably and have affected the application of these frameworks. Terms like ‘e-system’, ‘digital health’, ‘information system’, ‘health information systems (HIS)’ and those specific to human resources, such as ‘Human Resource Management Information System (HRMIS)’, ‘Human Resource Information Systems (HRIS)’ and ‘HRH-IS’ are often used interchangeably. This leads to misalignment, especially in selecting and applying the appropriate guidelines in healthcare to address the correct problem.^6,7,8,9^

Another limitation lies in the generic nature of many existing models. Some frameworks are overly broad, assessing diverse domains and indicators across the wider digital health ecosystem. They require substantial adaptation to fit LMIC health system contexts, before being narrowed more deeply to the inner workings of specific health domains. On the other end of the spectrum, certain models have been tailored for LMIC health systems, often benefiting from extensive international donor funding and research support. However, these tailored frameworks also have limitations. They usually prioritise specific diseases or health areas, reflecting the funder’s priorities, such as the President’s Emergency Plan for AIDS Relief (PEPFAR) which prioritises human immunodeficiency virus (HIV) and/or acquired immunodeficiency syndrome (AIDS). This overlooks the multi-faceted and interconnected nature of complex domains such as HRH. This was corroborated in a recent study examining LMICs in the African region, which questioned whether most countries are truly benefiting from the current wave of technological innovation or merely replicating disconnected, externally driven solutions.^8^

Where efforts were made in the localisation approach of these digital health frameworks, this has, in turn, presented new and different challenges. Most localisation efforts rely on subject matter experts (SMEs). These frameworks are adapted by experts who lack the necessary background, resources or expertise in domains like HRH, including those for monitoring health professionals’ education, leading to results that are either overly general or very technical, which limits their applicability, use and sustainability.

Consequently, there is limited evidence of digital health maturity models specifically designed to address the complexity of HRH-IS in African LMICs. Existing approaches often require substantial adaptation and lack alignment with workforce lifecycle management, including education, deployment and retention.

### Rationale for selecting a scoping review

Given the limited evidence on digital health maturity models, frameworks or tools specifically developed for the HRH domain, a scoping review was selected as the most appropriate method for this study. Scoping reviews are particularly suited for examining emerging fields that are characterised by diverse implementation approaches, unclear conceptual boundaries, definitions and terminologies and limited synthesis of existing literature in a geographic region. A scoping review approach also helps identify current trends shaping practice, highlights common characteristics, indicators, domains and methodologies used across frameworks, and uncovers existing knowledge gaps and lessons learned.^10^ This methodological choice aligns with the focus of the study being investigated.

### Objectives

This scoping review aimed to:

- Map existing digital health and HIS frameworks, models and tools relevant to HRH-IS in LMICs.
- Identify and synthesise key domains and approaches used to assess digital system maturity.
- Examine their relevance and applicability to HRH-IS within the African context.
- Inform the development of a context-appropriate HRH-IS maturity model.

## Methods

This scoping review was conducted following the framework developed by Arksey and O’Malley and further refined by Levac et al.^11,12,13^ The framework outlines five stages for conducting a scoping review: (1) identifying the research questions; (2) identifying relevant studies or literature; (3) selecting the relevant studies or literature; (4) extracting and analysing the data; and (5) summarising, synthesising, and reporting the results. The reporting and manuscript of this scoping review were drafted according to the Preferred Reporting Items for Systematic Reviews and Meta-Analyses extension for Scoping Reviews (PRISMA-ScR).^14^ Details on the items included in the PRISMA-ScR checklist are presented in Online Appendix 1.

### Operational definitions of terms

For this scoping review, the terms framework, model, maturity model, assessment tools, toolkit and guideline were used interchangeably and often in overlapping ways in the digital health and HIS literature.^4,15,16^ As such, these terms were treated as overlapping concepts, and they were further interpreted according to their purpose, structure and intended use to ensure consistent record screening, data extraction and synthesis.

#### Stage 1: Identification of the research questions

Identification of the research question ‘*What systems, tools and/or models are available for assessing the maturity of national capacities to track health professions education in the context of human resources for health development?’* was guided by the objective of the study.

#### Stage 2: Identification of relevant studies or literature

**Search strategy:** We conducted a comprehensive literature search across PubMed, EBSCO-hosted databases (including MEDLINE and Index to Nursing and Allied Health Literature [CINAHL]) and Google Scholar. Grey literature was identified through institutional repositories and websites, including WHO Institutional Repository for Information Sharing (IRIS), World Health Organization Regional Office for Africa (WHO AFRO) Health Observatory, World Bank Open Knowledge Repository, Ministries of Health websites and relevant partner organisations such as Global Fund, PEPFAR, United States Centers for Disease Control and Prevention (US CDC), United States Agency for International Development (USAID) and Department of Defense (DoD). No strict publication-date restriction was applied. However, priority was given to peer-reviewed literature published between January 2010 and December 2025, while no date restriction was applied to grey literature.

For searches on electronic databases, a search string and search frame was developed that combined multiple search terms; it was developed through discussions in conjunction with co-authors C.D.C. and H.K.K The search frame presented in Online Appendix 2 included a combination of keywords and subject headings across relevant domains such as HRH or workforce, informatics and information systems, their adaptability, implementation success and outcomes, specifically within LMICs in Africa. The full search strings used for PubMed, EBSCO-hosted databases and Google Scholar are presented in Online Appendix 2. Co-authors C.D.C. and H.K.K. tested to ensure that the selected search terms aligned with relevant vocabularies in databases such as MeSH (MEDLINE), EMBASE and CINAHL, ensuring pertinent studies with different and synonymous vocabularies were captured.

A grey literature search was conducted by co-authors C.D.C. and H.K.K. using a structured approach and guided by the review question, search framework and predefined eligibility criteria. The search focused on reports, national and global strategic documents and guidelines. Institutional websites and online repositories were systematically reviewed. Where search functionalities were available, keywords informed by the search frame presented in Online Appendix 2 were applied to identify relevant materials. Retrieved documents were screened using the same inclusion and exclusion criteria applied to peer-reviewed literature.

All search results from bibliographic databases, and grey literature sources were imported into Mendeley reference management software and duplicates were removed. A reference list of included studies was generated and subsequently screened for their eligibility and to identify any additional sources that met the eligibility criteria.

**Inclusion criteria:** The inclusion criteria comprised both peer-reviewed and grey literature that focused on digital health within broader health systems and those that were linked to HRH development in LMICs in Africa. Studies were also considered eligible if they addressed related areas such as conceptual frameworks, models or descriptive indicators relevant to the design, development or evaluation of information systems, including digital health and HRH information systems. Given the limited availability of HRH-specific maturity models, broader digital health frameworks were also included because they demonstrated conceptual relevance to HRH information systems including prior adaptation and implementation in African LMICs or comparable regional contexts. Relevance was determined based on the framework’s ability to assess capabilities applicable to workforce functions. In addition, documents that contributed to identifying key domains, sub-domains, or indicators for assessing the maturity of information systems, particularly those used in workforce management life cycle, planning, education and deployment, were included. Detailed inclusion criteria are presented in Box 1.

BOX 1 Inclusion criteria.

**Focus:** Research; peer-reviewed articles and grey literature mentioning digital health, tools, models, guidelines, and frameworks within broader health system contexts and demonstrating relevance to workforce development in LMICs in Africa.

**Conceptual relevance:** Articles and grey literature, including reports that included conceptual frameworks, maturity models, tools, guidelines or descriptive indicators related to the design, development, implementation or evaluation of digital health or HRH information systems.

**HRH linkages:** Research that described systems or strategies for health workforce planning, management, education, deployment or tracking within digital or information system environments.

**System maturity assessment:** Research, including country implementation reports, that contributed to identifying or evaluating key domains, sub-domains or indicators used to assess the maturity of systems tracking health professions education.

**Target population and stakeholders:** Research that included perspectives from policymakers, researchers, implementers or institutions involved in digital health and HRH, particularly within national or sub-national health systems.

**Geographic and contextual scope:** Research that primarily focused on LMICs in Africa; however, studies from high-income countries were also included if their findings or frameworks, models, guidelines or tools demonstrated clear applicability to LMIC settings.

**Language:** Only studies published in English and with full-text availability were considered.

**Time frame:** No strict timeframe restriction was implemented, but peer-reviewed articles published between 2010 and 2025 were prioritised, while grey literature had no time limit.

LMIC, low- and middle-income countries; HRH, human resources for health.

**Exclusion criteria:** Studies were excluded if they were not available in English. Additional exclusions included studies that focused exclusively on high-income countries without relevance to LMICs, those not related to HRH, digital health or maturity models, and non-empirical publications such as editorials, opinion pieces, blog posts or news articles.

#### Stage 3: Study and literature screening and selection

A total of 3128 records were identified from bibliographic databases and grey literature sources. These identified records were further subjected to a two-stage screening process.

In the first stage, titles and abstracts were independently screened by co-authors C.D.C. and H.K.K. using the predefined inclusion and exclusion criteria to identify potentially relevant studies. In the second stage, full-text versions of potentially relevant studies and works of literature were retrieved and assessed for eligibility. Screening decisions were compared and discussed between reviewers C.D.C. and H.K.K. Disagreements regarding record inclusion were resolved through discussion and consensus. In cases where consensus could not be reached, A.I.O. was consulted to make a final decision.

Forty-one records were selected for final review and systematic analysis as illustrated in Figure 1.^17^

**FIGURE 1:**
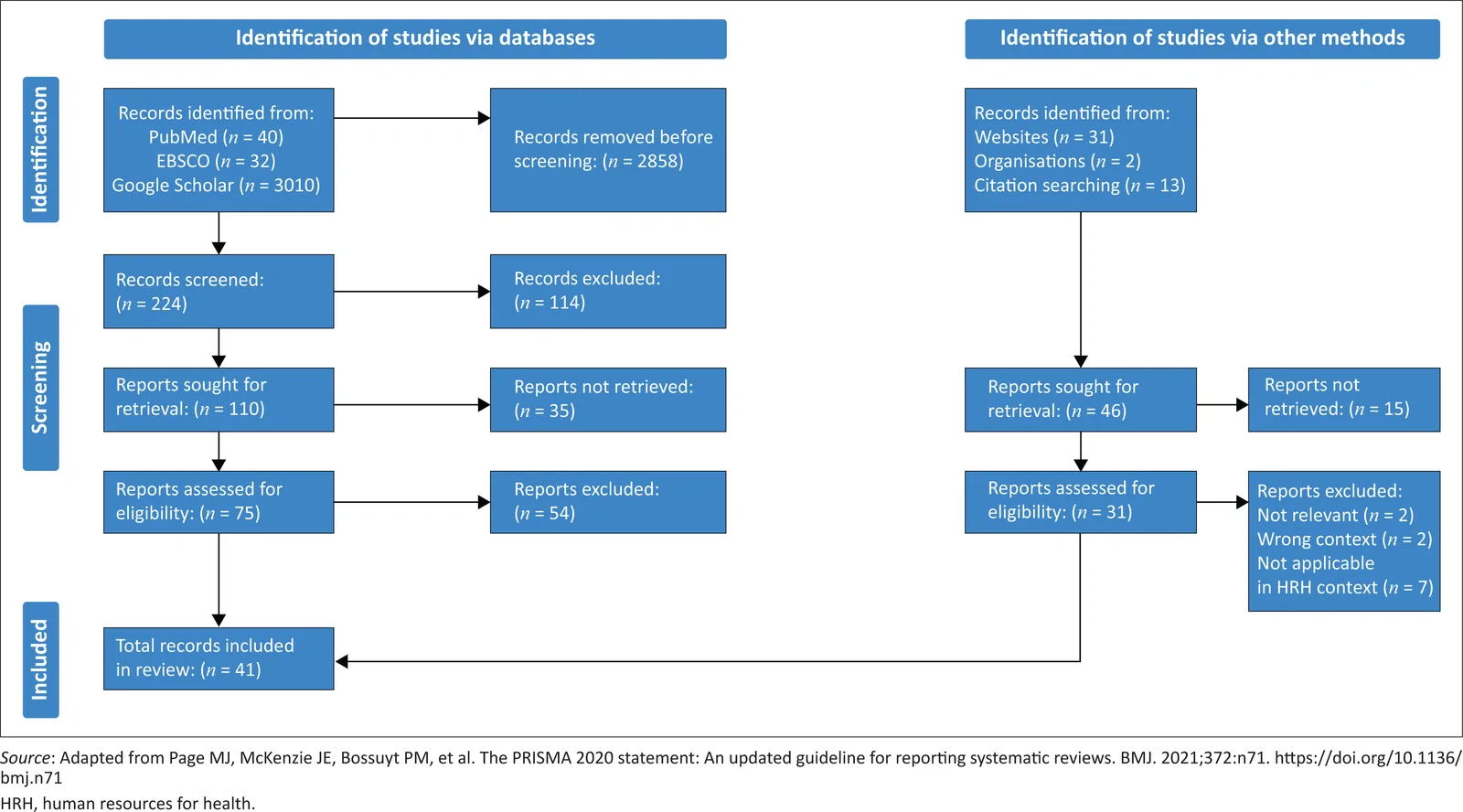
Flow diagram depicting the selection of sources of evidence.

#### Stage 4: Data extraction

A bespoke data extraction form (Online Appendix 3) was developed and used to extract relevant information from the selected records systematically. The extracted data were primarily descriptive and included both bibliographic and grey literature metadata and content related to the review question. Key elements captured in the data extraction form included: author(s), year of publication, country or income setting of the study, study type and details of the framework, model, or tool presented. Other elements included information on domains, components and other features relevant to the applicability of information systems, digital health and HRH information systems in LMICs.

#### Stage 5: Data analysis and synthesis of results

Data were analysed using descriptive mapping and thematic synthesis. Extracted frameworks, models and tools were categorised by type, such as maturity models, assessment toolkits, and strategic frameworks. Also extracted were the model or tool’s originating information, their scope of application, details on their adaptability and relevance to the HRH domain. A thematic analysis on emerging themes was conducted. This analysis involved identifying recurring domains, subdomains, indicators and system capabilities, across the included frameworks, models and tools. These extracted concepts were iteratively grouped into higher-level categories relevant to HRH-IS maturity. Where new themes emerged, additional thematic categories were created. Lessons from the development and application of maturity models, including insights from country-level implementation, were also synthesised.

### Ethical considerations

Ethical clearance to conduct this study was obtained from the North-West University Research Ethics Committee (NWU-HREC) (No. NWU-00240-25-S1).

## Results

Of the 41 records included for the review, only one record explicitly addressed an HRH maturity framework: the PEPFAR HRIS Assessment Framework. However, this framework was donor-driven and leaned towards a specialised health programme. The PEPFAR HRIS framework makes substantial reference to the HRH Action Framework, which was developed in 2006 and its application in LMICs in Africa fell outside the temporal scope of this review. Most of the remaining records focused on broader but related concepts, including digital health, HIS frameworks, maturity models, assessment tools, HRH registries and implementation resources.

As presented in Table 1, nearly half of the records (49%) were peer-reviewed publications, papers (*n* = 20) and one case study. These studies were conducted mostly in LMIC regions in Africa (*n* = 12), followed by those with a global scope (*n* = 4), LMIC and low-income countries (LI) combined (*n* = 1), upper-middle-income (UMI) (*n* = 1) and high income (*n* = 2).

**TABLE 1:** Summary of records included in the review.

Category	Number of records	Percentage of total	Scope and implementation region	Income classification
Peer reviewed records	20	48.8	Global scope: 4 records Sub-Saharan Africa: 4 records (including one covering 46 of 54 countries and another covering 8 countries) Nigeria-specific: 3 records Nigeria and Liberia: 1 record Ghana: 1 record South Africa: 1 record Other LMI and/or LMICs: 4 records (with one record a multi-country study on Kenya, Oman, Mozambique, Philippines, Indonesia, and another across 10 countries) Australia and Canada: 1 record US, UK, Canada, EU (Multinational): 1 record	LMIC: 12 records LI and LMIC combined: 1 record Upper middle-income (UMI): 1 record High-income countries (HIC): 2 records Global (mixed contexts and/or non-specific): 4 records
Global frameworks, guidelines, strategic roadmaps and other related resources such as digital taxonomy and classification	6	14.6	Global scope: 6 records	Global scope: 6 records
Global digital health benchmarking framework or assessment tools and related resources	2	4.9	Global scope: 2 records	Global scope: 2 records
Tools, maturity models, toolkits in health and applicable within the context of HRH development including technical reports of their application.	13	31.7	Global scope: 5 records LMIC: 5 records, Uganda: 2 records and Ghana: 1 record	Global scope: 5 records LMIC scope: 8 records

EU, European Union; HIC, high-income countries; HRH, human resources for health; LI, low-income countries; LMIC, low- and middle-income countries; LMI, low- and middle-income (used interchangeably with LMIC where applicable); UMI, upper middle-income; UK, United Kingdom; US, United States.

A further 20% represented global frameworks, guidelines and strategies for digital health and HIS with a global scope (*n* = 8). The remaining 13 records consisted of HIS assessment toolkits, digital health and maturity assessment toolkits, and implementation guides, along with technical resources tailored to the implementation of specific health systems and programmes. The studies were conducted across different contexts, with five records having a global scope and eight records focused specifically on LMIC settings.

Table 2 presents the 18 identified and extracted models and frameworks, or tools, that were relevant and applicable within the context of HRH development in LMICs. These records were not all HRH-specific; rather, they varied in scope, ranging from generic process maturity models and national digital health strategy toolkits to HIS interoperability, informatics readiness and HRH-disease-focused assessment tools. The extracted records excluded global guidelines, which in some cases were conceptualised using either one or a combination of multiple existing models. Furthermore, we also extracted other conceptual frameworks that were either adapted from other recognised models or frameworks, tested and adapted for implementation, leveraging localised context in LMICs.

**TABLE 2:** Frameworks, models and tools synthesised from the scoping review.

S/N	Framework, model or tool name	Description	Developer or source	Scope	Application region	Year implemented	Domains assessed	Maturity or scoring approach	Relevance to HRH-IS maturity	Notes and adaptability
1	Capability Maturity Model Integration (CMMI)^4,18^	A process and performance improvement framework that was originally developed for software engineering. Now, it is widely applied across industries, guiding organizations in developing and optimizing processes to improve quality, efficiency, and risk management.	Developed by the Software Engineering Institute (SEI) at Carnegie Mellon University; maintained by CMMI Institute (now part of ISACA).	Organizational processes, systems engineering, project management, quality assurance, and service delivery.	Model has been adapted and applied across different industries globally, including health. It served as a guiding framework for the development of other models.	1990s	Project management Engineering Process management Service delivery Quality assurance Risk management Supplier management	5 maturity levels: Initial Managed Defined Quantitatively Managed Optimizing	Highly relevant as a conceptual backbone for HRH-IS maturity. Offers a structured, staged model for investigating, localizing, assessing and improving processes, governance, data management, and system optimization.	Though not health-specific, CMMI’s staged approach has influenced many maturity models (including WHO, PAHO, DHPMAT-MM). Adaptable to HRH-IS in identifying and mapping relevant domains as well as a maturity or scoring approach.
2	WHO-ITU National eHealth Strategy Toolkit^19,20,21,22^	A toolkit developed to support governments and other stakeholders in developing a national eHealth vision and strategy, action plan, and evaluation mechanism.	WHO & ITU	Global scope and adaptable to LMICs and others.	Framework has guided the development of other models and tools.	2012	Leadership and governance Strategy and Investment Legislation, policy and compliance Workforce Standards and interoperability Infrastructure Services and applications	Strategy development roadmap (not numeric scoring)	Provides the foundation for a national digital health ecosystem, which is relevant for HRH-IS planning and integration to maximize service delivery and attain UHC.	Widely used reference; contains domains that are relatable and can be adapted to national HRH-IS contexts as part of broader digital health planning and integration.
3	Digital Health Profile and Maturity Assessment Toolkit Maturity Model (DHPMAT-MM)^4,22^	A model developed by WHO and ITU that stems from the CMMI model. It serves as a metamodel to unify various digital health maturity frameworks and guide digital health system assessment over time. It combines a digital health profile and a digital health maturity assessment.	WHO & ITU	Global scope and adaptable to LMICs and others.	Referenced implementation and piloting in pacific.^16^	2019	ICT infrastructure Digital tools Interoperability and readiness for information sharing Enablers of trust and adoption (Governance, funding, strategy, workforce capacity, literacy, stakeholder engagement) Quality Improvement, Monitoring, Measurement & Evaluation (QIME)	5-level proficiency scale adapted from CMMI Basic Controlled Standardized Optimised Innovative	Highly relevant to HRH-IS maturity. Model evaluates digital backbone, governance, workforce capabilities, interoperability, and continuous quality improvement, which are key to HRH information system development and maturity.	Model reinforces the adaptability of CMMI, including the maturing or scoring approach. Model also has domains that have relatable enablers within the context of HRH development.
4	ISHO Toolkit (Informatics-Savvy Health Department Toolkit)^24,25,26^	ISHO toolkit was designed to assess the informatics readiness at both national and site or organisational and/or facility levels, towards developing HIS strategies and a roadmap to strengthen informatics capabilities.	Public Health Informatics Institute (PHII)	Global and LMIC scope	References indicate adaptability and application in Nigeria and Zimbabwe.	2024 and 2025	Vision, Policy, and Governance Skilled workforce Effective information systems	6-level proficiency scale adapted from CMMI. Absent Initial Managed Defined Measured Optimized	Although focused within the HIV/AIDS domain, the toolkit emphasizes the strategic, governance, and workforce dimensions of informatics readiness, which are transferrable to an HRH-IS adoption and institutionalization.	Offers structured self-assessment whose domains are adaptable to HRH-IS contexts. In addition, it reinforces the use of the CMMI maturity or scoring approach.
5	Conceptual Framework for Strengthening eHealth Systems for UHC^25^ *(Adapted from informatics-savvy health department framework)*	A conceptual, strategic framework developed for leveraging eHealth to support universal health coverage.	Ojo A et al.	Sub-Saharan Africa; LMICs in Africa	N/A	2021	Governance & Leadership Strategy & Investment Infrastructure & Services Health Workforce & Capacity Building	Conceptual (no numeric scoring); structured as a strategic roadmap	Highlights HRH and workforce information as key enablers within eHealth for UHC. This is relatable to HRH-IS maturity whilst considering other enablers such governance, capacity and workforce data.	Regionally contextualized framework: complements WHO–ITU toolkit with adaptations from the informatics-savvy health department framework and tailored to Sub-Saharan African (SSA) challenges - low resources, fragmented HIS, capacity gaps).
6	Modified European Commission’s Digital Competency Framework^27^	A structured framework designed to assess digital competence and modified for healthcare settings to evaluate digital readiness of health professionals.	European Commission; adapted by Shiferaw et al. (2020)	Adaptable to LMICs	North-West Ethiopia	2020	Information/Data literacy Content creation Communication Problem solving, Safety	8-level proficiency scale	Focuses on digital skills of healthcare professionals, which is a critical HRH dimension of HRH-IS maturity.	Useful for integrating capacity-building and workforce readiness aspects into HRH-IS maturity models.
7	MAPS Toolkit (MHealth Assessment and Planning for Scale)^19,28,29^	A toolkit designed to assess readiness of mHealth interventions across multiple enablers and preparedness for scale-up and sustainability.	WHO, UN Foundation, Johns Hopkins University, and partners	Global and adaptable to LMICs	Reference to implementation in LMICs, including Ghana and Senegal	2018	Groundwork Partnerships Financial health Technology & architecture Operations, and Monitoring & evaluation	Uses guiding questions and maturity progression stages for scalability	Provides a structured lens to evaluate readiness and scalability of digital health solutions, including workforce-related systems.	Adaptable to HRH-IS particularly the enablers for scale-up and sustainability planning, per LMIC settings.
8	HIS Interoperability Maturity (HISIM) Toolkit^22,30,31,32^	A toolkit developed for governments and other stakeholders to determine domains for interoperability and the necessary levels of maturity, towards achieving national HIS goals.	MEASURE Evaluation	LMICs	LMIC with case studies in Uganda and Ghana	2019	Leadership & Governance Human Resources Technology	5 stages of maturity: Nascent Emerging Established Institutionalize Optimized	Directly relevant for HRH-IS integration and interoperability with other systems, including enablers such as workforce and governance. Can guide in identification of relevant indicators to assess in the development of the HRH-IS maturity model.	Strong for governance and HRH aspects, including their sub-domains, which can be adapted for HRH-IS maturity assessments.
9	Stages of Continuous Improvement (SOCI) Toolkit^22,33,34^	Toolkit for countries or organizations to assess, plan, and prioritize interventions to strengthen an HIS.	MEASURE Evaluation	Global and adaptable to LMIC	-	2019	Governance & Leadership Management and Workforce ICT Infrastructure Standards and Interoperability Data Quality and Use	5 maturity stages Emerging/Ad Hoc Repeatable Defined Managed Optimized	Highlights enablers for national level HIS, including relatable indicators across both the toolkit’s core domains and sub-domains that are relevant to the institutionalization and sustainability of an HRH-IS.	Useful to track incremental adoption and domains and sub-domains that align to HRH-IS integration and sustainability with continuous improvement cycle.
10	Routine HIS Rapid Assessment Tool^22,35,36^	A rapid assessment tool to assist in identifying gaps and weaknesses at service delivery points or facilities.	WHO and MEASURE Evaluation	LMICs	N/A	2018	Management and governance Data and decision support system Data collection and processing Data analysis dissemination and use	Checklist & scoring (binary and/or qualitative indicators)	Highlights domains and sub-domains relevant to workforce planning and training, training capacity and other areas.	Strong in localized indicators at service delivery level. It has transferable domains that can be adapted within the context of HPE and HRH development.
11	Performance of Routine Information System Management (PRISM) Toolkit^22,37^	Framework and series of tools to diagnose strengths and weaknesses of routine health information systems, focusing on data quality and use.	MEASURE Evaluation	LMICs	N/A	2019	Driven by 3 determinants: technical, organisational and behavioural.	No formal maturity or proficiency scale defined	Highlights data quality and use as core component of this toolkit, which are also central to HRH-IS effectiveness.	Contains an organizational and behavioral assessment tool, which relates to health professionals and may have some transferable indicators.
12	PEPFAR HRIS Assessment toolkit^28,38^	A toolkit designed to assess HRIS functionality within HIV/AIDS programmes, focusing on workforce data use for programme delivery.	HRH2030 Program (USAID, Palladium, consortium partners)	LMICs	References implementation in Malawi and Zambia	2017	This toolkit assesses two sets of domains: 8 HRIS functions (*Pre-service Education, Registration and Licensure, Staffing Gaps and Needs, Payroll Information, Personnel Actions, In-service Training, Workforce Exit/Attrition, Health Worker Registry*) and another 8 HRIS capacities (*Technology Infrastructure, Decentralization, Use of Standards, Data Quality, Sustainable Financing, Human Capacity, Interoperability and Data Use*).	5-level proficiency scale adapted	Although the toolkit is focused on a particular programme area, it highlights domains relevant for tracking the lifespan of health professionals.	Although the toolkit was developed for HIV/AIDS, it includes relevant domains that can be adapted and expanded by drawing on existing models and knowledge from HRH-specific records. This ensures that the relevant domains, which incorporate the perspective of health professionals including their education, are included.
13	HRH Optimization Tool for Family Planning (HOT4FP), for ART (HOT4ART) and for Primary Health Care (HOT4PHC)^29,39^	These tools were designed to assess HRH availability and functionality, including recommending distribution strategies through task shifting and sharing to support a particular programme area such as family planning, HIV/AIDS and selected Primary Health Care (PHC) services.	HRH2030 Program (USAID, Palladium, consortium partners)	LMICs	References were made to implementations in Malawi and Zimbabwe; however, these resources and adapted tools were unavailable due to recent reprioritization with US donor funds.	2021	Toolkits assessed the imbalances in HRH professionals required for the scale-up of disease areas and integration into the PHC. Specific domains include workforce numbers and HRH allocations, skill mix, task-sharing, service delivery workload.	FTE calculations comparing existing and required staffing	Highlights HRH professionals’ gaps for integrating specific disease into PHC.	Although primarily focused on HRH in family planning, ART, and primary health care, they also offer additional dimensions for HRH-IS assessment.
14	Digital Pandemic Preparedness Assessment (DPPA) Tool^23^	This toolkit was developed to assess and map gaps in countries’ digital health ecosystems for pandemic preparedness and response, including vaccination roll-out and integration with existing systems.	Digital Square (USAID, Gates Foundations and partners)	LMICs	LMICPiloted and tested in Côte d’Ivoire, Ghana, Nigeria, Sierra Leone, and Togo	2021–2022	Human capacity Infrastructure Investments & funding Standards & interoperability Data capture & use Governance and policy	Qualitative and Quantitative scoring guided through instructions and interpretation	Relevant to HRH-IS maturity as the toolkit assesses digital health system readiness, including integration of health information systems, data interoperability, and governance capacity for pandemic contexts.	Provides contexts or domains from different countries that can contribute to articulating relevant domains and sub-domains for HRH-IS maturity.
15	Information Systems for Health (IS4H) Toolkit^40^	Toolkit developed to guide countries and other stakeholders in strengthening their HIS towards achieving UHC.	PAHO/WHO (Pan American Health Organization / World Health Organization)	LMICs	North and South America	2021–2022	Governance Data policy Monitoring & Evaluation (M&E) Interoperability Digital transformation strategy	No numeric scoring; structured templates and qualitative guidance	Supports HRH-IS maturity with relatable domains such as governance, policy, strategic roles, M&E frameworks, and data system interoperability.	Toolkit is modular and is widely adaptable, especially the M&E and interoperability domains.
16	Digital Health Systems Maturity Assessments (DHSMA)^24^ *Not a formal framework*	A conceptual model, partly adapted from WHO ITU and ISHO, and used to identify barriers and facilitators of maturity assessments, focusing on enabling conditions and stakeholder alignment.	Phiona Vumbugwa et al.	Global and adaptable in LMICs	Applied in 10 African countries	2024	Leadership and Governance Health Financing Health information Health workforce Service delivery Medical supplies	Qualitative insights (no numeric scale)	Highlights real-world barriers and strategic priorities for maturity of national digital health systems, which are valuable and can be tailored to HRH-IS maturity approaches.	Empirically grounded, with country experiences, and can serve as lessons learnt from LMICs, which can optimise the development and adaptation of an HRH-IS maturity tool in LMIC contexts
**Other inter-related models**										
17	Global Digital Health Index(GDHI) and MaturityModel^22,41^	Index for tracking, monitoring, and evaluating progress in digital health technology at the country level, across the 7 components of the WHO-ITU eHealth strategy framework.	World Health Organization (WHO)	-	-	2016	Leadership & governance, Strategy & investment Legislation, policy and compliance Workforce Standards & interoperability Infrastructure Services and applications	5 phased maturity scoring, but across 23 indicators with a mix of qualitative and quantitative measures and country benchmarking capabilities.	Highlights real-world approaches to establishing benchmarking capabilities across countries.	Can contribute to identifying comparable domains and sub-domains relevant to an HRH-IS maturity framework in LMICs.
18	WHO Digital Health Atlas^15,22^	A global, open-source registry platform designed to catalogue, visualize, and coordinate digital health projects, serving as a technology investment registry.	World Health Organization (WHO)	N/A	N/A	2018	N/A	N/A	This resource can serve as a complementary source of existing digital health implementation in LMICs.	-
19	Human resources for health information system: minimum data set for health workforce registry.^42,43,44,45,46,47^	Although not a framework, it is relevant to HRH-IS as it highlights data points relevant to the life cycle of a health professional	World Health Organization (WHO) National and/or subnational	LMICs	Nigeria and Zimbabwe	2022	Highlights multiple domains and enablers relevant to the broader HRH development context. Domains include governance, project coordination, intervention mapping, interoperability classification, scale-up and monitoring.	N/A	This resource offers a new perspective because it contains context specific to HRH development and implementation learning across LMICs.	This resource provides the localisation framing for an HRH-IS maturity framework. It is pivotal and can drive adaptability and localisation to HPE, HRH and within LMIC settings.

Note: Please see the full reference list of the article Oaiya AI, Christmals CD, Kipruto HK. Digital health maturity models for Human Resources for Health Information Systems in low- and middle-income countries in Africa: A scoping review. J Public Health Africa. 2026;17(1), a2010. https://doi.org/10.4102/jphia.v17i1.2010, for more information.

ART, antiretroviral therapy; BHCPF, Basic Health Care Provision Fund; CMMI, capability maturity model integration; DHPMAT-MM, Digital Health Profile and Maturity Assessment Toolkit Maturity Model; DPPA, digital pandemic preparedness assessment; FTE, full-time equivalent; GDHI, global digital health index; HIS, health information system; HISIM, health information system interoperability maturity; HOT4ART, HRH optimization tool for antiretroviral therapy; HOT4FP, HRH optimization tool for family planning; HOT4PHC, HRH optimization tool for primary health care; HRH, human resources for health; HRH-IS, human resources for health information system; HRIS, human resources information system; HWR, health workforce registry; ICT, information and communication technology; IS4H, information systems for health; ISHO, Informatics-Savvy Health Department Toolkit; ITU, International Telecommunication Union; LMICs, low- and middle-income countries; M&E, monitoring and evaluation; MAPS, MHealth assessment and planning for scale; MDS, minimum data set; MoH, Ministry of Health; PAHO, Pan American Health Organization; PEPFAR, President’s Emergency Plan for AIDS Relief; PHC, primary health care; PHII, Public Health Informatics Institute; PRISM, performance of routine information system management; SEI, Software Engineering Institute; SOCI, stages of continuous improvement; SSA, sub-Saharan Africa; UHC, Universal Health Coverage; USAID, United States Agency for International Development; WHO, World Health Organization; S/N, serial number; HIV, human immunodeficiency virus; AIDS, acquired immunodeficiency syndrome; UN, United Nations; HR, human resource; US, United States; HPE, health professions education; ISACA, Information Systems Audit and Control Association.

Regarding the domains in the included models and frameworks, recurring themes encompassed governance, vision and leadership, workforce capacity and organisational readiness, financing and sustainability, infrastructure and technology, standards and interoperability, data quality and use, monitoring and evaluation, and service delivery. These domains were consistently represented across a wide range of digital health, HIS and HRH-related frameworks. This suggests a broad consensus on the capabilities required for the development, institutionalisation and long-term sustainability of information systems.

The maturity approaches varied across the 18 included models and frameworks. Forty-four per cent (*n* = 8) of the models and frameworks used staged maturity scales. Of these, 7 (39%) applied either a five- or six-level Capability Maturity Model Integration (CMMI) style maturity scale, while 6% (*n* = 1) used an eight-level proficiency scale. The remaining frameworks relied on other approaches, including qualitative or roadmap-based assessment without formal maturity scoring, checklist-based methods and mixed qualitative and quantitative scoring. This also indicates a strong preference for staged maturity assessment approaches within the digital health and HIS literature.

## Discussion

### Restating the research purpose

This review highlights a critical gap in the availability of dedicated HRH-IS maturity models in African LMICs. While numerous digital health and HIS frameworks exist, very few are specifically designed to address the complexity of workforce information systems. As a result, countries often rely on adapting broader frameworks that may not fully capture HRH-specific requirements.

### Definitions and taxonomies of digital health and workforce

#### Digital health definition

We found that over the past decade the definitions and classifications of digital health have continued to evolve. In a 2020 review, Farhad Fatehi identified 95 distinct definitions of ‘digital health’, with 30 drawn from journal articles and 39 from other sources.^8^ However, as time progressed, there has been a gradual shift towards looking to the WHO as the leading authority and unifying reference point for digital health terminology.^19,20,21,48^ This trend was consistently reflected in most records reviewed.

WHO defines digital health as:

[*T*]he systematic application of information and communications technologies, computer science, and data to support informed decision-making by individuals, the health workforce, and health systems, to strengthen resilience to disease and improve health and wellness. (p. 7).^48^

The concept of digital health is built upon earlier terms such as eHealth and mHealth, a subset of eHealth.^19,20,21^ While eHealth is ‘the use of information and communications technology in support of health and health-related fields’, mobile health (mHealth), focuses more on mobile and wireless devices. mHealth is ‘the use of mobile wireless technologies for public health’.^19,20,21^ The broader term digital health has been framed as ‘a concept encompassing eHealth (including mHealth), as well as emerging areas such as big data, genomics and artificial intelligence’.^19,20,21^

#### Human resources and human resources for health interpretation

The human resource (HR) and HRH domains have also experienced misconceptions and similar transformations. HR is critical for every organisation and is operationalised through Human Resource Management (HRM), which encompasses the entire employee life cycle: selection, recruitment and hiring, training, compensation, performance tracking and the management of workplace relationships to ensure optimal delivery. Technologies developed for HR, such as HRIS or electronic HRM (e-HRM), are designed to acquire, store, manipulate, analyse, retrieve and distribute pertinent information about an organisation’s workforce.^46^ These systems support HR management functions and extend to broader HRIS applications, including payroll.

However, HR is often confused with HRH, and this misconception has affected the design, implementation and delivery of HRH-related interventions in Africa. As a result, many programmes have been poorly developed and implemented, driving further fragmentation rather than promoting integration and sustainability.^49^

Similar to the evolving definition of digital health, the WHO is clarifying how HRH-IS and health workforce registries (HWR) are conceptualised, developed and integrated in the large health system.^47^ Increasingly, emphasis is being placed on coordinating data use across countries to ensure comparability at regional and continental levels, guided by the WHO and its regional systems; the National Health Workforce Accounts (NHWA).^28,42,43,45,47^

Although HRH-IS and HWR are often used interchangeably and confused with HRIS, their applications differ within the health system, which is inherently complex and involves multiple organisations with overlapping functions and roles. WHO clarified the relationship between HRH-IS and HWR by also emphasising the commonalities of data sets collected on health professionals through a minimum data set (MDS),^47^ further re-emphasising that HWR are a subset of HRH-IS, which has similar functions to an HRIS. However, the nuances differ depending on the country, and these technologies in the HRH context need to be designed to complement existing public service structures rather than operate in parallel or in competition. For example, in some LMICs, the terms HRH-IS and HWR perform similar functions, serving as the single authority for health workers, and are better institutionalised at both sub-national and federal levels, stimulating data use for workforce planning and policy formulation. This is largely because workforce regulations are viewed as core governance functions requiring centralised oversight to ensure standards and accountability. These workforce regulation roles, also including recruitment and in some cases performance management, are often HRIS-related and remain within public sector labour entities, reflecting their administrative nature and alignment with broader civil service management processes rather than health sector–specific governance.

### Frameworks, models and tools for human resources for health information systems and applicable in low- and middle-income countries

Our scoping review identified 18 models.^4,5,8,15,18,19,20,21,22,23,24,25,26,27,28,29,30,31,32,33,34,35,36,37,38,39,40,41,42,43,44,45,46,47,48,49,50,51,52,53,54,55^ Among these, only one was directly related to HRH: the PEPFAR HRIS Assessment Tool. This tool itself had its challenges. Although the tool included valuable HRH lifecycle functions such as pre-service education, registration and licensure, and health worker registry functions, the tool was developed within the context of the HRH Action Framework^28^ to support workforce strengthening for HIV and/or AIDS programmes.

Most of the other frameworks and models were developed to assess broader digital health, HIS, or informatics readiness. While these models provide foundational domains with available literature on their adaptability, they do not fully capture the complexities of the HRH context needed for HRH planning and management, governance and labour market analysis in African LMICs. Key components for tracking health workforce lifecycle – such as pre-service education tracking, registration and licensure and workforce attrition – and their associated complexities, which require coordination among multiple institutional entities including ministries of health, ministries of ICT, health training institutions, and regulatory councils, are either sparsely addressed or fully omitted. Furthermore, we identified that the reprioritisation of funding from the United States government created a significant void, as several resources and tools that may have been tested and validated on the continent have either been lost or are no longer accessible.

### Emergent domains and maturity approach

Most frameworks, models and in some cases, tools, were conceptualised around the enhanced CMMI framework.^4,5,8,15,18,19,20,21,22,23,24,25,26,27,28,29,30,31,32,33,34,35,36,37,38,39,40,41,42,43,44,45,46,47,48,49,50,51,52,53,54,55^ The CMMI model is structured around two key dimensions: capability levels, domains and sub-domains, which assess the performance of specific process areas and maturity levels, also commonly referred to as the scoring approach that evaluate the overall growth of an organisation’s processes. Together, these provide a measure of capability maturity – the extent to which desired process or features are defined, managed, and optimised over time.

#### Emergent capability: Domains and sub-domains

Although most of the models and frameworks identified in this review were not primarily developed for the HRH domain, they demonstrated clear similarities and relevance to strengthening HRH, if appropriately adapted. Table 3 presents model capabilities observed and synthesised across six areas that promote health systems integration, efficiency, ownership and sustainability. These capabilities are critical for evaluating the implementation of digital systems and are transferable to the HRH context in LMICs.

**TABLE 3:** Synthesised domains relevant to HRH-IS maturity model development.

Capabilities or domains	Description	Possible alignment with health professionals within HRH context
Governance and Stewardship, Leadership and Policy	Encompasses the strategic direction, legislation, regulation, compliance, stewardship, and accountability mechanisms.	Existence of a national digital health strategy or blueprint with HWR referencing, Existing national policies on privacy, security and confidentiality; Existence of national HRH policies referencing HRH-IS or HWR; Existence of national HRH policies or plans referencing data use from HRH-IS or HWR for strategic plan development; Existence of governance structures such as HRH units embedded in national and sub-national MoH across different administrative levels; Emerging accountability frameworks for workforce reporting; Existence of national HRH reports such as workforce profiles.
Health Workforce and Organizational Capacity	This domain focuses on the institutional human resource capital required to adopt and sustain an HRH-IS. It encompasses the workforce skills, training, readiness, willingness, human factors, and institutional capacity.	Existing HRH focal points at ministries and/or HRH units; Emerging capacity in workforce information management; Existing training programmes in HRH data literacy; Existing frameworks on HRH registry management, and use of digital HRH tools.
Financing and Investment	This domain focuses on funding for HRH expanding into resource allocation, funding models, sustainability, and cost-effectiveness of national digital health initiatives and streamlined into HRH.	HRH-IS initiatives were largely donor-funded, and the decoupling of USAID may have created a void. Areas to explore include existing digital health funding mechanism, existing direct funding mechanism or models such as BHCPF in Nigeria, and initial advocacy for integrating HRH-IS into national health budgets.
Data Architecture, Infrastructure, Technology, and System Interoperability	This encompasses the data points for HRH-IS that promotes local workforce planning and management, including comparability across other LMICs. This domain also includes the HRH-IS ICT infrastructure, its operations, interoperability with other HIS, standards, security, and privacy.	Existing HRH-IS operational plans encompassing HRH data points built on and expanded from recommended MDS; Existing and recommended basic ICT infrastructure in HRH units; Existing MoH networking infrastructure; Existing policies and practises on managing MoH databases either localised standalone HRH databases.
Data Quality, Information Systems, Analytics and Integration in Service Delivery	This domain focuses on the linkages between data architecture and technology, with sources and custodians of health workforce data positioned at other levels. Specifically, it assesses data availability, accuracy, completeness, management, and exchange, as well as the functionality of registries, directories, and other systems. Furthermore, it examines the capacity to apply advanced analytics for evidence generation and decision-making.	Existing sources of HRH data (payroll, council, training institutions); Previous development of HRH registries and /or directories; Existing HRH data quality assurance practices or frameworks; Existing structures or data pipelines for workforce analytics for workforce planning; Initial use of HRH dashboards or workforce monitoring apps; pilot applications for tracking deployments or training; Functional adoption of HRH tools at subnational levels.
Equity, Trust, and Adoption and Impact	This domain focuses on the access, utilization, acceptability, digital literacy, safety, user experience, readiness to share information, and enablers of trust of an HRH-IS.	Emerging discussions on data privacy and trust; Use of HRH data in decision-making; Evidence of impact on policy formulation and workforce allocation.

BHCPF, Basic Health Care Provision Fund; HWR, health workforce registry; HRH, human resources for health; HRH-IS, human resources for health information system; HIS, health information system; ICT, information and communication technology; LMICs, low- and middle-income countries; MDS, minimum data set; MoH, Ministry of Health; USAID, United States Agency for International Development.

#### Emergent maturity scale: Scoring approach

Similarly, the maturity scales were largely structured around five or six stages, also adapted from the CMMI improvement approach. Most frameworks assessed maturity using five phases ranging from *Emerging/Ad Hoc*, through *Repeatable, Defined* and *Managed*, to a final stage of *Optimised*. Others localised the maturity scale to the context of the specific health programme area or disease area, depicting stages that progressed from *Basic*, through *Controlled, Standardised* and *Optimising*, to *Innovating*. Although most maturity models used five stages, we found that the specific labels, growth description and scaling structures varied; nevertheless, they were consistently adapted to fit the context of the assessment. For an HRH-IS maturity framework, the maturity scale should be adapted to reflect the realities of the workforce lifecycle and their enablers across multiple domains within the LMIC context.

### Implications for policy and practise

This scoping review identified a critical gap in context-specific HRH-IS maturity frameworks, models, or tools for the African LMICs context. This poses significant implications for health policy reform and practice, including system design and implementation. The continued reliance on generic digital health frameworks that are largely donor-driven has resulted in poor local adaptations. This often results in fragmented and unsustainable HRH-IS implementations, leading to a waste of scarce resources and further compounding existing workforce challenges in the region.

In the wake of recent funding disruptions and the reprioritisation of external support across most LMICs, countries in Africa are increasingly confronting a new reality. This has led to LMICs beginning to rethink and reshape their health systems. In this context, this scoping review presents an opportunity for diverse stakeholders, particularly policymakers, workforce practitioners, implementers, health system managers, digital health architects, labour and health workforce regulatory institutions, training institutions and development partners to rethink and rebuild HRH-IS as a critical component of the health system through the lenses of integration, ownership, financing, governance, trust and sustainability.

The findings from this review provide a structured basis to strengthen HRH-IS through the application of six synthesised and inter-related domains that reflect the realities in most LMICs in Africa. These domains work together to create synergy across systemic enablers specific to HRH. In doing so, they support the development of HRH-IS that are tailored to local contexts, integrated within existing health system structures, financed and aligned with national health system priorities.

For example, policymakers and decision-makers can leverage relevant domains to guide strengthening governance structures, including aligning HRH-IS with national strategies, and ensure sustainable financing. While workforce practitioners ensure that HRH data collected and used in the system are relevant, high quality and effectively used for workforce planning and policy development, digital health architects can apply the domains related to data architecture and interoperability to design an HRH-IS that meet required standards and integrates with existing HIS. Civil society organisations (CSOs) can engage with relevant domains related to equity, trust and adoption to promote transparency, accountability and inclusive use of workforce data.

The findings from the scoping review highlight the need for a deliberate shift from fragmented, donor-driven approaches towards a collective, integrated, context-specific and country-owned process. By leveraging the six synthesised domains, policymakers and system actors can strengthen the design, implementation, and sustainability of HRH-IS, ensuring alignment with national priorities and improving the effectiveness of workforce planning and health system performance.

### Limitation

This review has several limitations. Firstly, the inclusion of diverse document types, including grey literature, introduces heterogeneity in the evidence base. Secondly, no formal quality appraisal was conducted, consistent with scoping review methodology. Thirdly, the restriction to English-language sources may have excluded relevant studies. Finally, the reliance on adaptable frameworks limits the ability to draw conclusions about the effectiveness of HRH-IS-specific maturity models.

## Conclusion

This scoping review demonstrates a clear gap in dedicated HRH-IS maturity models in African LMICs. While only one HRH-specific tool was identified, a broader ecosystem of digital health and HIS frameworks provides a strong foundation for adaptation. The six synthesised domains offer a preliminary basis for developing a context-specific HRH-IS maturity model. Future research should focus on translating these domains into measurable indicators and validating the model through country-level implementation.
